# Pyelo-hepatic abscess caused by staghorn stone infection: a case report

**DOI:** 10.1186/s13256-023-04173-9

**Published:** 2023-10-23

**Authors:** Octavio J. Salgado, Katherine S. Pesantes-Barros, Beatriz C. Rosales, Lizette Espinosa-Martin

**Affiliations:** 1 School of Medicine, Universidad Católica de Cuenca, Av. Las Americas Y Humbolt, Cuenca, 010101 Azuay Ecuador; 2 Department of Nephrology, University Hospital of Maracaibo, Maracaibo, 4001 Zulia Venezuela

**Keywords:** Case report, Liver abscess, Staghorn calculus, Renal infection

## Abstract

**Background:**

The most common source of pyogenic liver abscess is biliary tract infection. Other less common routes include the spread of bacteria from distant foci. However, direct extension of a perinephric infection focus to the liver is extremely rare.

**Case report:**

The patient was a non-diabetic, immunocompetent, 29-year-old woman of mixed race ancestry with a history of recurrent urinary tract infections who was referred to our hospital because of an ultrasound-detected liver abscess. She was initially treated with metronidazole for 20 days at the referring institution for suspected amebic abscess without improvement. On admission to our center, she was febrile and complained of a dull right upper quadrant pain. A POCUS ultrasound suggested a pyogenic abscess, probably from a staghorn calculus infection. She received meroperem and amikacin for 22 and 10 days, respectively. Repeat hemocultures showed no growth, but urine cultures were positive for Proteus sp. Complete remission of clinical and imaging findings was observed under antibiotics. The patient was referred to the urology outpatient clinic to discuss the option of radical nephrectomy.

**Conclusion:**

This case underlines the high morbidity of staghorn calculi.

## Background

Liver abscesses consist of a collection of pus within the liver parenchyma and are generally an uncommon complication. Except in endemic regions, where the amebic etiology may be predominant [1], most liver abscesses are secondary to bacterial infection [2].

The most common source of pyogenic liver abscesses is biliary tract infection due to obstruction and inflammatory conditions [3]. Other less common routes of hepatic invasion include the spread of bacteria from distant foci (apendicitis, diverticulitis, etc.) to the liver parenchyma through the portal circulation [4] or via the hepatic artery in the setting of bacteremia [5].

More rarely, a hepatic abscess may result from penetrating (infected liver biopsies) or blunt (biliary stenting in the case of malignancy) hepatic trauma [3], from miscellaneous causes such as extension from empyema of the gallbladder [6], from a subphrenic abscess or due to cholecystogastrocolonic fistula [7]. Known risk factors for liver abscess include age > 65 years, male sex, diabetes mellitus, malignancy, alcoholism, cirrhosis and liver transplantation [8]. However, direct extension of a perinephric focus of infection to the liver is extremely rare [1]. Only 3 cases have been described in a comprehensive review of the literature. We report for the first time a liver abscess as an extension of a primary renal infectious focus originating from a right-sided staghorn calculus in a young, nonimmunocompromised woman.

## Case report

The patient was a nondiabetic 29-year-old female of mixed racial ancestry with a history of recurrent urinary tract infections. She denied other comorbidities. She was referred to our hospital from another healthcare facility with a sonografically confirmed diagnosis of liver abscess and staghorn stone in the right kidney. She had been treated with intravenous metronidazole for 2 weeks before referral for a suspected amebic liver abscess without improvement. On admission, she complained of moderate pain in the right upper quadrant of the abdomen and of a mild to moderate fever for the past 3 weeks. The patient denied any history of renal colic, the passing of urinary stones, or any gastrointestinal symptoms. On physical examination, the patient was in apparently preserved general condition, axillary temperature of 38.2 °C, no conjunctival jaundice, marked tenderness in the right upper quadrant of the abdomen on deep palpation. Blumberg negative. Laboratory analysis showed mild anemia, leukocytosis of 12.7 K/µL. Serum biochemistry parameters, particularly aminotransferases and total bilirubin, were within normal range. Serum creatinine was 0.9 mg/dL. HIV 3rd Generation ELISA antibody testing  was negative. Urinalysis showed moderate bacteriuria. Urine cultures were positive for Proteus spp. with a colony count of 100,000 CFU/mL. Strain serotyping was not performed. Blood cultures obtained prior to the initiation of antibiotic therapy were negative. Serial microscopic examinations of concentrated stool samples did not reveal any ova or vegetative forms of any parasite.

**Fig. 1:**
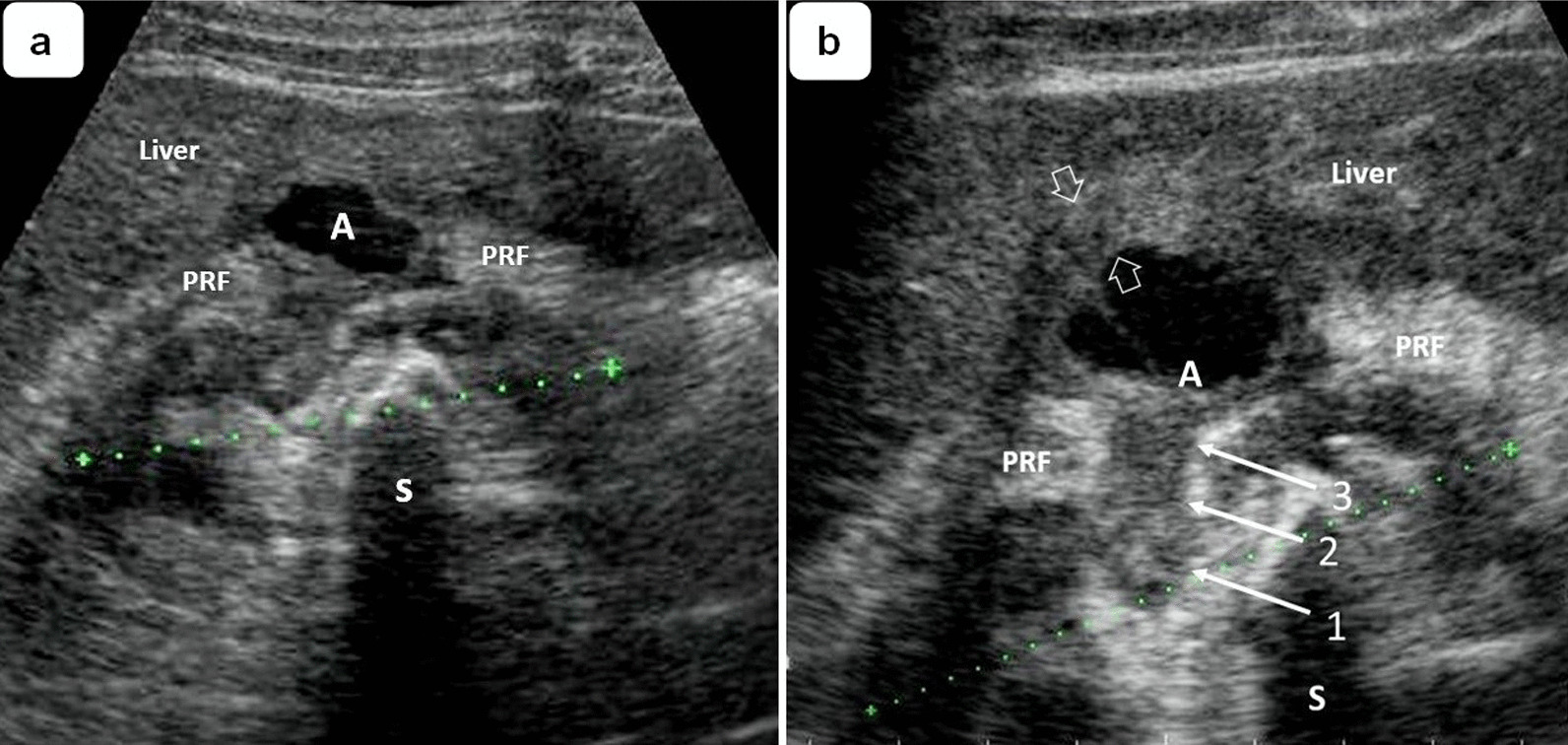
**a** Reduced right kidney (longitudinal axis = 7.5 cm) with parenchymal thinning and staghorn stone occupying pelvis and calyces. An anechoic collection of 2.7 × 1.6 cm is seen within the hepatic parenchyma adjacent to the kidney, surrounded by a hypoechoic rim (hollow arrows) corresponding to a hepatic abscess [A]. **b** Close-up view of the liver lesion showing the most probable route of infection [arrows 1, 2, and 3], which originates from the middle third of the right kidney. S: Acoustic shadow. PRF: Perirenal fat

**Fig. 2:**
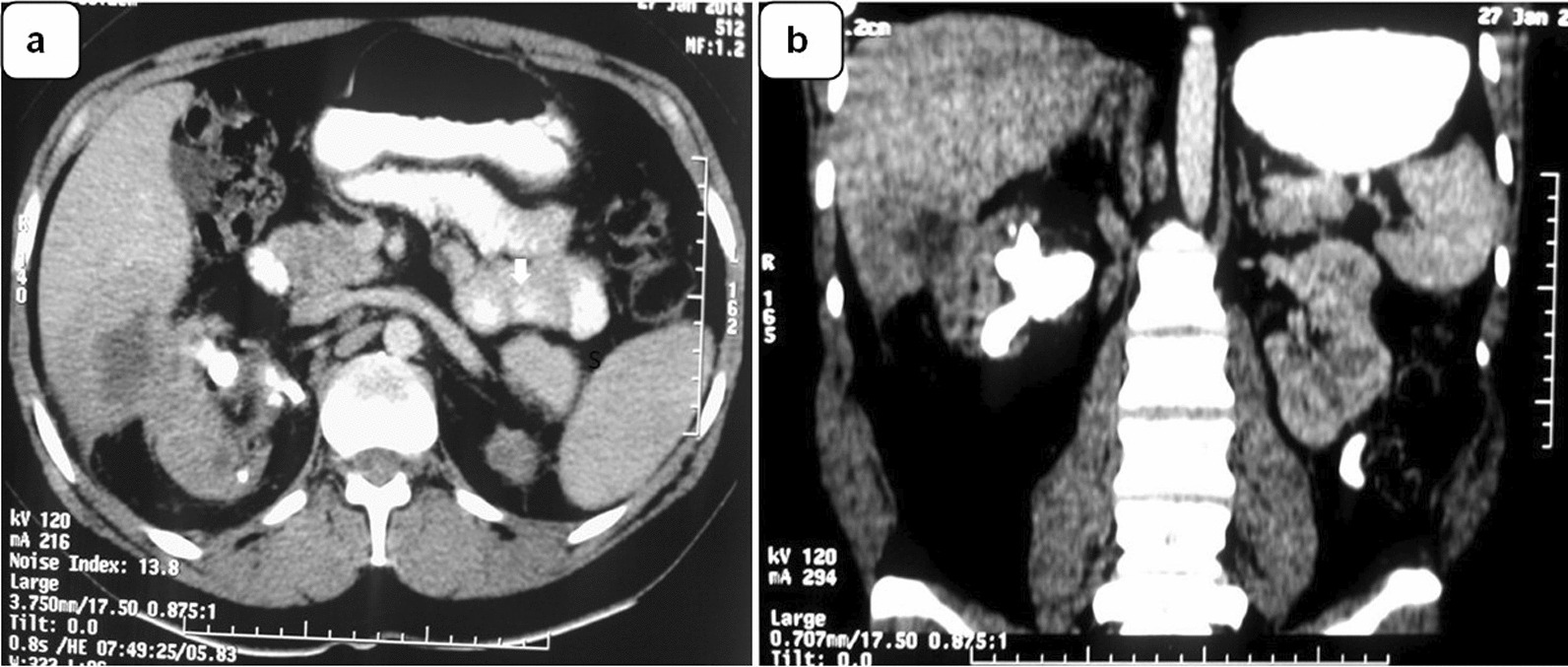
**a** Transverse CT scan showing the liver abscess surrounded by an area of intermediate density corresponding to the hypoechoic rim seen on ultrasound. **b** Coronal CT scan shows a staghorn stone in the right kidney and a hepatic abscess most likely located in segment VI of the liver forming a heterogeneous mass involving perirenal fat and the adjacent renal parenchyma

Abdominal ultrasound of the right upper quadrant showed a solitary hypoechoic lesion measuring 2.7 × 1.8 × 1.5 cm surrounded by an enhancing rim located in the right hepatic lobe segments adjacent to the right kidney (Fig. 1a). The kidney was decreased in size (longitudinal axis = 7.5 cm), accompanied by parenchymal thinning. In projection to the central zone, a staghorn calculi occupying the renal pelvis and calyces (acoustic shadows) can be appreciated. Zooming in on the area of the lesion (Fig. 1b), it was possible to follow the path of the infection process [arrows 1, 2, and 3], which appears to have started in the middle third of the right kidney [arrow 1], passing through the renal capsule [arrow 2], the perirenal fat [PRF], and finally penetrating Glisson's capsule [arrow 3] and the liver parenchyma. The gallbladder was reported to be unremarkable. No dilatation of the biliary tree was observed. The left kidney measured 9.6 cm in its longitudinal axis and its appearance was echographically within the normal range. The cross-sectional CT (Fig. 2a) showed a hepatic abscess, most likely in segment VI of the liver, surrounded by an area of intermediate density corresponding to the hypoechoic rim seen on ultrasound. The coronal CT scan (Fig. 2b) showed a staghorn stone in the right kidney. In both views, the perirenal fat was part of the inflammatory area, forming a heterogeneous mass involving the adjacent renal parenchyma and hepatic abscess.

The patient was treated empirically with a combination of 1 g/IV meropenem every 8 h and 500 mg/IV amikacin every 12 h. As the results of the urine culture and the antibiotic susceptibility test showed good sensitivity of the isolated bacteria against both antimicrobials, the ongoing therapy was continued for 22 days and 10 days, respectively.

**Fig. 3:**
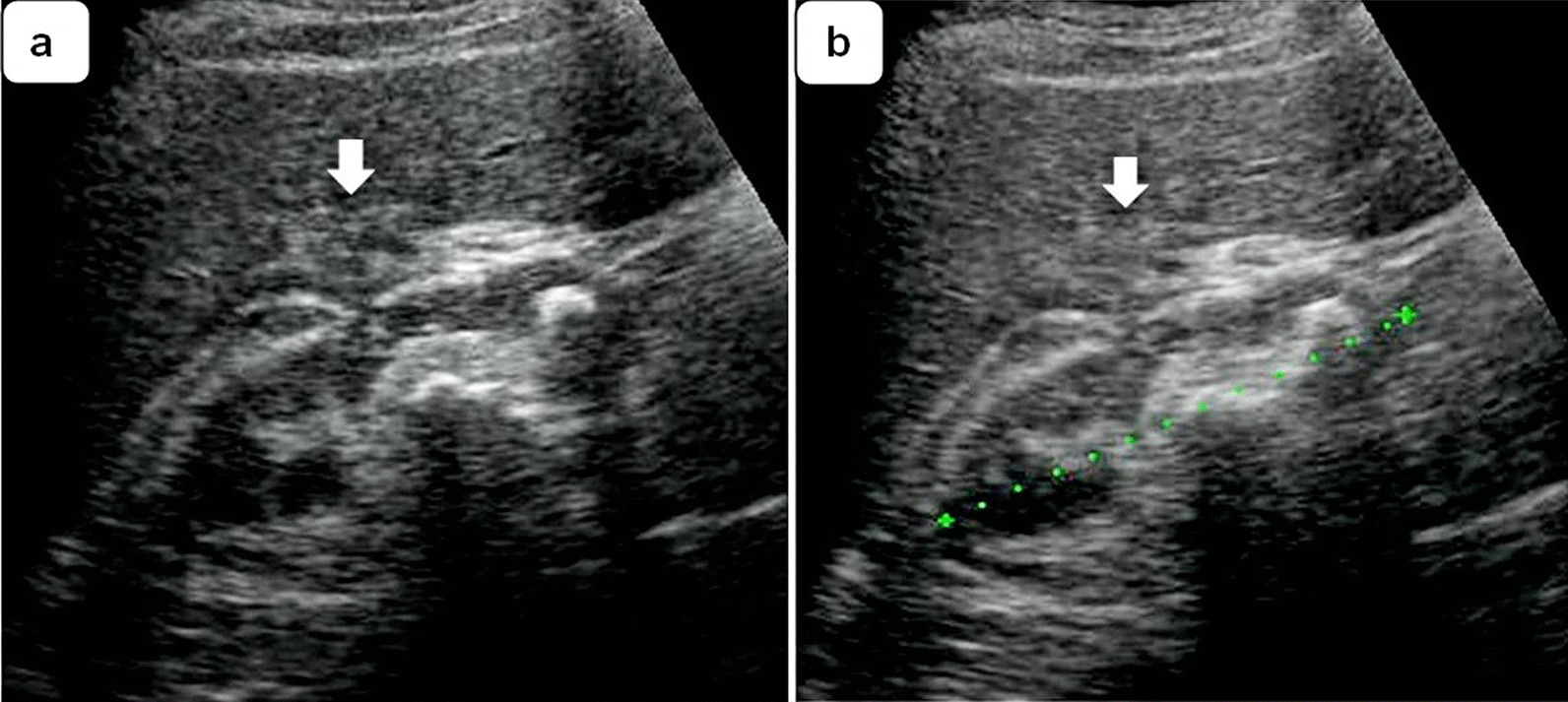
Appearance of the liver abscess (arrows) (**a**) on day 16 and (**b**) on day 22 after initiating antibiotic therapy showing complete resolution

Complete remission of symptoms was achieved after the first week of therapy. A repeat ultrasound scan after 3 weeks of antibiotic treatment showed complete resolution of the abscess (Fig. 3). The patient was discharged from hospital without symptoms and referred to the urology outpatient clinic for further management.

## Discussion

Lithiasis is a   common cause of renal infection [9]. The pathomechanism of infection is partly dependent on the type of stone. Calcium and uric stones are usually noninfectious and result from an imbalance between factors that promote or inhibit urine crystallization [10]. In patients with these types of calculi, infection may occur secondarily after long-standing obstruction, leading to bacterial overgrowth associated with urinary stasis [11].

In contrast, struvite and calcium carbonate-apatite stones, known as infection stones, are caused by repeated infections with urea-splitting bacteria and account for 10–15% of all stones [12]. Infected stones may occupy either part or all of the pelvis and calyceal groups, resulting in the formation of a partial or complete staghorn calculus. Cultures of fragments of staghorn calculi taken from both the surface and the core have shown that bacteria can hide in the fissures within the stones, meaning that the calculi themselves act as a reservoir for infection [13].

In most cases, regardless of the source, renal infections remain confined to the kidney. If left untreated or inadequately treated, and depending on the immunological status of the host, staghorn calculi may cause recurrent infections, pyonephrosis and, more seriously, xanthogranulomatous pyelonephritis (XGPN) [14]. Peri- or paranephric abscesses are often the starting point for spread through contiguity with adjacent anatomical structures. Rupture of peri- and perinephric abscesses into the peritoneum is relatively common [15]. Extension to neighboring intestinal segments has been reported to give rise to the formation of reno-duodenal [16] and nephrocolonic fistulae [17]. Other less commonly reported spreading sites of peri- or perinephric abscesses include the retroperitoneum [18], psoas muscle [19], vena cava, and renal vein causing infectious thrombosis [20]. The involvement of more distant structures, such as the spleen [21], prostate [22], and lungs and bronchi, with the formation of nephron-bronchial fistulae [23] has also been occasionally observed.

As mentioned above, extension of a renal infectious focus to the liver is extremely rare and has been reported in only 3 cases (Table 1). In 2 of 3 cases [24, 25], the etiology of pyonephrosis was urinary obstruction due to urolithiasis. In the remaining case [26], pyonephrosis and perirenal abscess developed as a complication of XGPN. No underlying staghorn stone was observed on imaging or histopathologic analysis of the excised kidney. Of note, in a series of 1000 cases, staghorn calculi were found in 48% of patients diagnosed with XGPN   [27]. The present case is the 4th case of a hepatic extension of a renal infectious focus and the first in which the primary infectious reservoir was a staghorn calculus.

**Table 1:** Cases of kidney infection with extensión to the liver reported in the literature

Author/year Ref. number	Patient’s age/sex	Infectious source of liver abscess	Isolated germ	Treatment	Outcome
Tanwar *et al.* 2013 [25]	25/F	Pyonephrosis due to obstruction by calculus	*E. coli*	Surgical	Favorable
Chung *et al.* 2008 [26]	43/F	Xanthogranulomathous Pyelonephritis	–	Conservative	Favorable
Kahan *et al.* 1972 [24]	69/F	Pyonephrosis due to obstruction by calculus	*E. coli*	Surgical	Favorable
Present report	29/F	Staghorn stone infection	*Proteus* sp	Conservative	Favorable

As shown in Fig. 1b, the infectious process appears to have started in the middle third of the kidney, passed through the renal capsule, erupted into the perinephric space and finally penetrated the liver parenchyma. The clinical and imaging features suggest that the present case may correspond to a focal XGPN with extension to the liver parenchyma. The hypoechoic rim (hollow arrows) surrounding the abscess is most likely an area of solid necrotic tissue originating from the central zone of the kidney and extending through the capsule at the lateral border of the kidney (Fig. 1b, arrows 1, 2 and 3). However, there is no histopathologic study available to confirm this diagnostic reasoning.

At the referring institution, the patient was initially treated unsuccessfully with intravenous metronidazole for 20 days under the diagnostic suspicion of an amebic liver abscess, which is the most common type of liver abscess in tropical endemic areas. On admission to our hospital center, the patient was started empirically on intravenous meropenem and amikacin as soon as the point-of-care ultrasound (POCUS) findings indicated a possible pyogenic liver abscess.

Urine cultures were positive for Proteus sp., which was sensitive to both antibiotics given. However, hemocultures showed no bacterial growth. The patient became asymptomatic on treatment with normal blood WBC. Ultrasound examinations on day 21 showed complete resolution of the lesion, respectively (Fig. 3). The patient was discharged asymptomatically and referred to the urology outpatient clinic for further management.

## Conclusions

This case provides new evidence of the high morbidity of staghorn calculi and the need to consider radical nephrectomy as a measure to prevent recurrent infection, especially when kidney damage is advanced and residual function is negligible. This case also highlights the importance of POCUS in the initial diagnostic work-up of hepatic and renal complications, as in the present case.

## Data Availability

All information is available in the clinical records archive of the University Hospital of Maracaibo, Venezuela.

## Abbreviations

CT: Computed tomography
WBC: White blood count
POCUS: Point-of-care ultrasound
